# Observation of Spin-Glass-like Behavior over a Wide Temperature Range in Single-Domain Nickel-Substituted Cobalt Ferrite Nanoparticles

**DOI:** 10.3390/nano12071113

**Published:** 2022-03-28

**Authors:** Gassem M. Alzoubi

**Affiliations:** Department of Physics, Faculty of Science, The Hashemite University, P. O. Box 330127, Zarqa 13133, Jordan; gassem@hu.edu.jo

**Keywords:** single domain, ferrite nanoparticles, Rietveld refinement, magnetization, spin-glass, LA model

## Abstract

In this study, single-domain ${\mathrm{Ni}}_{x}{\mathrm{Co}}_{1-x}{\mathrm{Fe}}_{2}O_{4}$ ferrite nanoparticles with 0≤x≤1 were hydrothermally prepared and characterized using X-ray diffraction, transmission electron microscopy (TEM), Fourier transform infrared spectroscopy (FT-IR), and vibrating sample magnetometry. According to the Rietveld refinement results, all of the prepared nanoparticles were single phase with spinel-type structures. Increasing the Ni content increased the average crystallite size and X-ray density while decreasing the lattice constant. According to the TEM observations, the nanoparticles were spherical in shape. The formation of a single-phase spinel structure with two lattices centered at tetrahedral and octahedral sites was confirmed by the observation of two absorption bands in all FT-IR spectra. Magnetization data showed that the prepared nanoparticles of all compositions were ferrimagnetic across the entire temperature range of 300 K to 10 K. Magnetic properties such as saturation magnetization, remanent magnetization, coercivity, magnetic anisotropy, and magnetic moments per unit cell were found to decrease with increasing Ni content. The big difference in $H_{c}$ of the x = 0, 0.25, 0.5, 0.75 ferrites between 300 K and 10 K suggested that these ferrite nanoparticles are truly single-domain nanoparticles. The small value of $H_{c}$ of the ${\mathrm{NiFe}}_{2}O_{4}\;\left(x=1\right)$ ferrite and its very weak temperature dependence suggested that this sample is in a multi-domain regime. The ZFC–FC curves revealed the existence of spin-glass-like behavior in these ferrite nanoparticles over the entire temperature range.

## 1. Introduction

Magnetic ferrites, a ceramic-like material, have received much attention in recent decades because of their fascinating magnetic properties, which can be used in a variety of electronic devices such as magnetic storage devices, gas-sensing devices, microwave devices, and a wide range of other biomedical applications [1,2,3,4]. Spinel ferrites, a type of magnetic ferrite, have a cubic spinel structure and belong to the space group Fd3m. The chemical formula of spinel ferrites can be represented by ${\mathrm{MF}}_{2}O_{4}$, where $M^{2+}$ and $F^{3+}$ are metal ions that represent divalent and trivalent metal ions, respectively [5]. The spinel unit cell is made up of eight formula units of ${\mathrm{MF}}_{2}O_{4}$ containing a total of 32 oxygen ions in a cubic closed packing (CCP) arrangement. In between the oxygen ions, there are two types of vacancies: 64 tetrahedral (A sites) and 32 octahedral (B sites). Only 8 of the available 64 A sites are occupied by cations, and only 16 of the 32 B sites are occupied by cations. Spinel ferrites have size-dependent magnetic properties that are primarily influenced by the synthesis method and cation distribution between the tetrahedral and octahedral sites.

Cobalt ferrite ${\mathrm{CoFe}}_{2}O_{4}$ is among the most important types of spinel ferrites because of its remarkable electrical and magnetic properties, which include high resistivity, high coercivity, and high magnetic anisotropy [6,7]. Controlled substitution can be used to tune the magnetic properties of cobalt ferrites. The magnetic characteristics of cobalt ferrite nanoparticles can be substantially altered by replacing hard magnetic Co^2+^ ions with soft magnetic Ni^2+^ ions in a controlled manner [5,8]. Nickel ferrite (${\mathrm{NiFe}}_{2}O_{4}$) is a soft magnetic material that crystallizes into an inverse spinel structure. It has a low coercivity, high saturation magnetization, and high resistivity [5,6]. Magnetic ferrites have been made using a variety of techniques, including sol–gel, coprecipitation, hydrothermal, and mechanical alloying [9,10,11,12,13].

The size dependence of ferrimagnetism in cobalt ferrite nanoparticles (CFNs) is one of their most exciting magnetic properties. From the largest to the smallest particle size, there are three major domain types: multi-domain, single-domain, and superparamagnetic domain. For CFNs, the multi-domain regime is formed when the size of the nanoparticles is greater than 40 nm [14]. When a particle’s size falls below 40 nm, it can no longer be split into domains and is locked in a single domain [14,15]. If the particle’s size is decreased to less than 20 nm, it will begin to change from ferrimagnetic to superparamagnetic at a temperature known as the blocking temperature ($T_{B}$). Even at low temperatures, thermal fluctuations in the superparamagnetic regime can easily exceed the magnetic anisotropy energy barrier of the nanoparticle and hence randomize its magnetic moment, resulting in degraded ferrimagnetism [5,12]. Thus, despite much research performed on cobalt ferrite, the manufacture of nanoparticles with the desired size and magnetic properties remains a challenge.

Spin-glass behavior has been observed in many magnetic ferrite nanoparticle systems [5,12]. The spin-glass state exists in magnetic nanoparticles primarily due to magnetic frustration and disordered spins at the nanoparticles’ surface. When the size of a nanoparticle is small enough, the surface atoms experience different environments than those in the nanoparticle’s core. There are various types of defects that may exist on the surface of the nanoparticle, such as dangling bonds and atomic vacancies. These defects result in uncompensated disordered spins at the nanoparticle’s surface [16]. Because the surface spins are randomly aligned, this state is commonly referred to as a spin-glass-like behavior state. The spin-glass behavior of magnetic nanoparticles is typically investigated experimentally using zero-field-cooled (ZFC) and field-cooled (FC) magnetization measurements. The shape of the ZFC–FC curves provides qualitative information about this state.

In recent decades, extensive research has been conducted on cobalt ferrite and its doped families. The average size of the nanoparticles produced in these research works was greater than 40 nm, resulting in a weak size dependency of their magnetic properties [10]. “This is due to the fact that the synthesis of ultra-small and monodisperse nanoparticles is regarded as a challenge in this field” [12]. Single-domain magnetic nanoparticles are of particular interest as they have high sensitivity and high spin reversal performance. This motivated us to investigate the connection between the size of these nanoparticles and their magnetic properties within the single-domain regime. The primary goal of this research was to optimize the hydrothermal recipe for making single-domain monodispersed ${\mathrm{Ni}}_{x}{\mathrm{Co}}_{1-x}{\mathrm{Fe}}_{2}O_{4}$ ferrite nanoparticles, as well as to characterize their structural, morphological, and magnetic properties. Understanding the magnetic characteristics of these stable ferrite nanoparticles and having a reproducible recipe for producing them in the single-domain regime is essential for using them in a variety of applications, including magnetic devices, chemical sensors, and biomedical applications [4,17].

## 2. Materials and Methods

In this study, the hydrothermal method was used to create ${\mathrm{Ni}}_{x}{\mathrm{Co}}_{1-x}{\mathrm{Fe}}_{2}O_{4}$ ferrite nanoparticles with 0≤x≤1 from high-purity metal nitrates. The synthesis procedure is well known and has already been thoroughly described in our previous publications [5,7,12]. To improve the crystallinity of the prepared ferrite nanoparticles, all samples were subjected to 6 h of high-temperature annealing at $700{\;}^{\circ }C$[9]. The crystal structures of the samples were investigated using X-ray diffraction (XRD). The XRD spectra were obtained using a Rigaku Ultima IV diffractometer with a Cu–K${}_{\alpha }$ radiation source (λ=1.5406 Å). The surface morphology and average particle size were examined using a JEOL 1200 transmission electron microscope (TEM). The analysis of the Fourier transform infrared (FT-IR) spectra provided information on the samples’ vibration bonds. The FT-IR spectra were recorded using a Bruker FT-IR spectrometer. At temperatures ranging from room temperature to 10 K, the magnetic properties were investigated using a Quantum Design PPMS integrated with a vibrating sample magnetometer (VSM). Magnetization data were collected in magnetic fields ranging from −25 kOe to 25 kOe.

## 3. Results and Discussion

### 3.1. XRD Analysis

**Figure 1:**
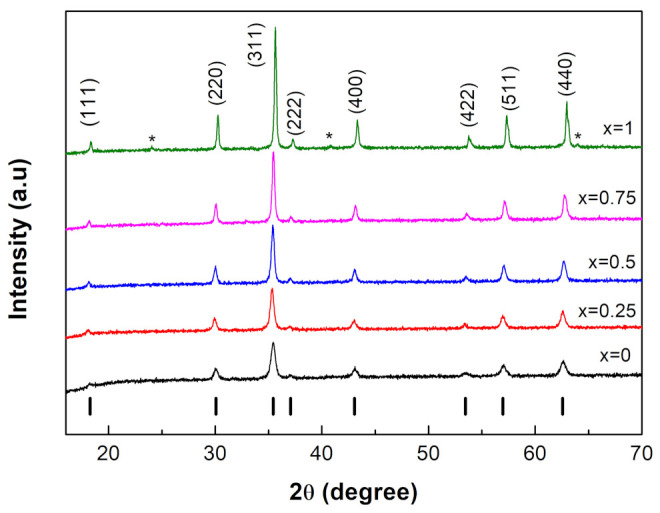
XRD patterns of the ${\mathrm{Ni}}_{x}{\mathrm{Co}}_{1-x}{\mathrm{Fe}}_{2}O_{4}$ spinel ferrites with 0≤x≤1. The black vertical bars represent the standard Bragg reflections of the space group Fd3m.

Figure 1 shows the XRD patterns of the ${\mathrm{Ni}}_{x}{\mathrm{Co}}_{1-x}{\mathrm{Fe}}_{2}O_{4}$ ferrites with different Ni content (0≤x≤1). The space group Fd3m was successfully used to index all XRD reflections, confirming the cubic spinel structure of the prepared ferrite nanoparticles. Samples with 0≤x≤0.75 were single phase, while that with x = 1 contained a trace of unknown impurities. All of the diffraction patterns in the figure corresponded to the well-known cubic spinel structure, which is represented by the vertical black bars at the bottom. The high peak intensity indicated a high degree of crystallinity in the nanoparticles produced.

By using the FullProf software to Rietveld-refine the XRD spectra, information about the cell parameters, grain size, X-ray density, and bond lengths was obtained [18]. “Following each refinement trial, the Rietveld fitting quality was evaluated using the reliability R-factors (expected $R_{\exp}$ and weighted profile $R_{\mathrm{wp}}$) and goodness of fit ($\mathrm{GoF}={\left(R_{\mathrm{wp}}/R_{\exp}\right)}^{2}$)” [7]. A satisfactory fit is usually obtained once these quantities reach their minimum values. Because both cobalt and nickel ferrites have an inverse spinel structure [7,12], all cations were assembled between tetrahedral (8a) and octahedral (16d) sites to maintain the stoichiometric composition of the ${\mathrm{Ni}}_{x}{\mathrm{Co}}_{1-x}{\mathrm{Fe}}_{2}O_{4}$ ferrites. The Wyckoff positions (8a, 16d, and 32e), atomic coordinates (x, y, z), and site occupancy (sof) used during Rietveld refinement of the ${\mathrm{Ni}}_{x}{\mathrm{Co}}_{1-x}{\mathrm{Fe}}_{2}O_{4}$ ferrites are shown in Table 1.

**Figure 2:**
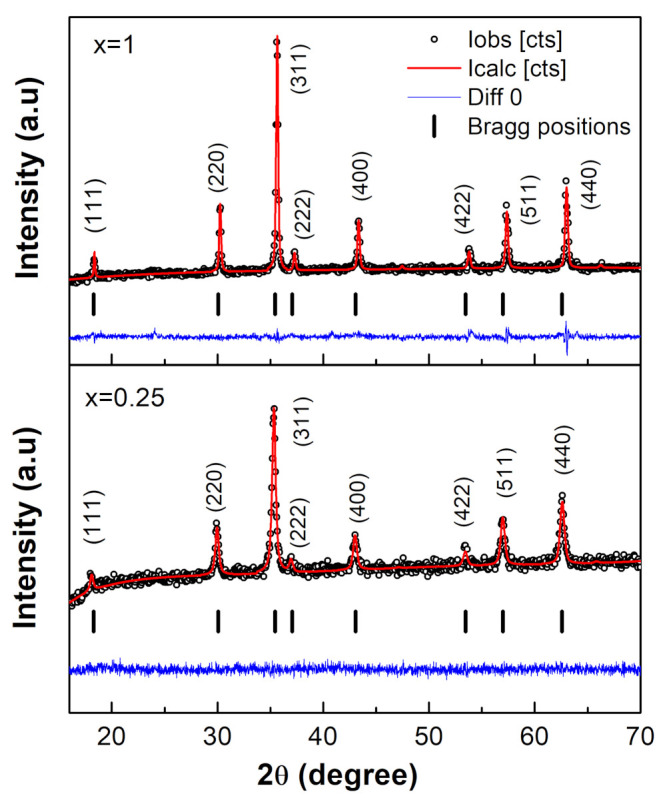
XRD powder pattern and Rietveld refinement of the ${\mathrm{Ni}}_{x}{\mathrm{Co}}_{1-x}{\mathrm{Fe}}_{2}O_{4}$ ferrites with x = 0.25 as a representative sample. The experimental data are represented by black circles, the calculated data by a red line, and the difference between the two by a blue line. Bragg positions are represented by black vertical bars.

A typical Rietveld refinement for ${\mathrm{Ni}}_{x}{\mathrm{Co}}_{1-x}{\mathrm{Fe}}_{2}O_{4}$ ferrites with x = 0.25 as a representative sample is shown in Figure 2. As shown in the figure, the observed data and the calculated data had great agreement. Table 2 summarizes the results of the refinement for this sample, as well as the other samples. “The three essential refined quantities were the oxygen positional parameter *u*, the experimental lattice parameter $a_{\exp}$, and the full width at half maximum (FWHM). The X-ray density $d_{x}$, the crystallite size D, and the bond lengths at the tetrahedral $R_{A}$ and octahedral $R_{B}$ sites were computed using these values” [12]. Oxygen anions form a cubic close packed (CCP) array of anions with an ideal u value of 0.25 Å in a perfect spinel structure [7]. The CCP’s regularity was determined by the u value. The oxygen anions in real spinel ferrites, on the other hand, are displaced from their optimal CCP positions, resulting in significant distortion from the ideal CCP structure and, as a result, a change in the value of u. The u value usually varies from 0.24 Å to 0.27 Å. Table 2 shows that the value of u was 0.2564 Å for ${\mathrm{CoFe}}_{2}O_{4}$ (x = 0) and 0.2565 Å for ${\mathrm{NiFe}}_{2}O_{4}$ (x = 1). The obtained values of u agreed with previously reported values of 0.2556 Å and 0.2594 Å for cobalt ferrite [19] and nickel ferrite [8,20], respectively.

**Figure 3:**
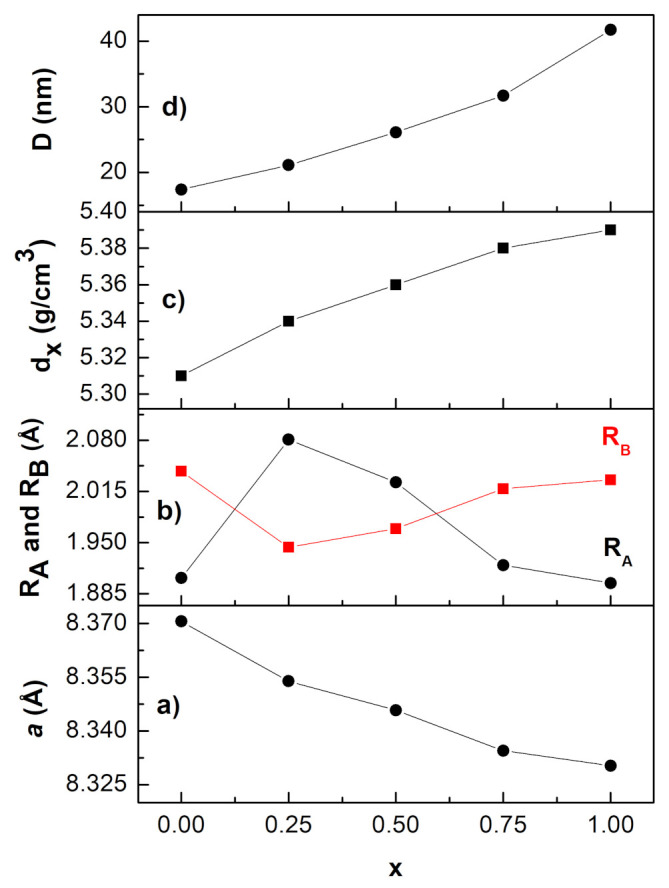
Variation of some structural quantities as a function of Ni content: (**a**) lattice constant, (**b**) the average bond lengths of $R_{A}$ and $R_{B}$, (**c**) the X-ray density, and (**d**) the crystallite size.

Figure 3a shows that the lattice constant decreased with the gradual replacement of Co ions by Ni ions, as predicted by Vegard’s law [21] when an ion of larger radius is replaced by an ion of smaller radius. As a result, as Ni content increased, the observed lattice constant ($a_{\exp}$) decreased from 8.3706 Å (for x = 0) to 8.3303 Å (for x = 1), as illustrated in Table 2. These obtained values of $a_{\exp}$ were consistent with previously measured values of 8.3730 Å [22] and 8.3688 Å [9] for cobalt ferrite and 8.3330 Å [20] and 8.3180 Å [9] for nickel ferrite, respectively.

Using the relationships given in [5], the average bond lengths at the tetrahedral ($R_{A}$) and octahedral ($R_{B}$) sites can be evaluated. Figure 3b shows the calculated $R_{A}$ and $R_{B}$ values for all compositions. Nickel and cobalt ions tend to favor octahedral sites, whereas iron ions can exist in both octahedral and tetrahedral sites. For an ideal substitution, where the Ni^2+^ ions of a smaller radius (0.69 Å) replace Co^2+^ ions of a bigger radius (0.76 Å) on the octahedral sites, the $R_{B}$ was predicted to decrease and the $R_{A}$ to remain unchanged. However, this trend was not followed in the prepared ferrites, suggesting that cation disorder may occur in the spinel structure of the prepared ${\mathrm{Ni}}_{x}{\mathrm{Co}}_{1-x}{\mathrm{Fe}}_{2}O_{4}$ ferrites, which in turn affects structural properties such as the bond length between cations. The behavior of the parameters $R_{A}$ and $R_{B}$ should be related to the behavior of the parameter u. As u changes, the anion sublattice expands or contracts, until the volumes of the A and B sites match the radii of the constituent cations [5].

Table 2 includes the measured values of X-ray density, $d_{x}$, for all ferrite compositions. As shown in Figure 3c, the density increased with Ni content. This behavior can be understood by remembering that the density of the crystal lattice is directly proportional to the molar mass and inversely proportional to the lattice constant. Because the molecular weights of Co and Ni are so close, the increase in X-ray density was mainly a result of a reduction in the unit cell volume caused by the introduction of smaller-sized Ni ions into the spinel structure.

The average crystallite size, D, of all ferrite samples was evaluated by measuring the full-width at half-maxima (FWHM) of the most intense peak (311) from the XRD pattern and by using the Scherrer equation [12]:

(1) $$D=\frac{K\lambda }{\beta \cos\theta }$$

where K, λ, θ, and β represent the shape factor (K=0.89 for spherical particles), the wavelength of the X-ray radiation (λ=1.5406 Å), the diffraction angle, and the FWHM measured in radians. Figure 3d depicts the crystallite size variation with Ni content for all ferrite compositions. As can be seen in the figure, the crystallite size increased as Ni content increased, most likely due to nickel’s tendency to interact with oxygen anions during grain growth. The crystallite size increased from 17.4 nm to 41.7 nm as x increased from 0 to 1. For the same ferrite composition, but prepared by the sol–gel method, the crystallite size increased from 36 nm to 58 nm as x increased from 0 to 1 [23].

### 3.2. TEM Characterization

**Figure 4:**
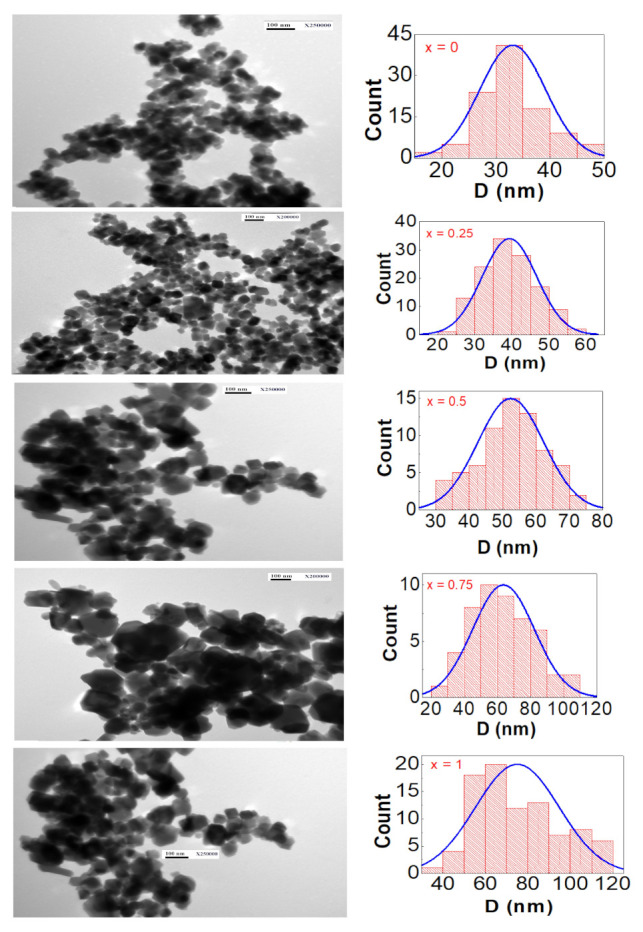
TEM images and particle size distribution histograms for the ${\mathrm{Ni}}_{x}{\mathrm{Co}}_{1-x}{\mathrm{Fe}}_{2}O_{4}$ spinel ferrites with 0≤x≤1. Starting at the top (x = 0, 0.25, 0.5, 0.75, 1).

The morphology and particle size distribution for all compositions of the ${\mathrm{Ni}}_{x}{\mathrm{Co}}_{1-x}{\mathrm{Fe}}_{2}O_{4}$ ferrites are shown in Figure 4. These TEM images were acquired at 250,000-times magnification. The majority of the particles in all TEM images appeared to be spherical, indicating that the recipe was effective at producing spherically shaped nanoparticles. Statistical analysis of at least 80 nanoparticles yielded the average particle size, D, of each sample from the corresponding TEM image. The obtained D values were plotted as a histogram, which was then fit with a Gaussian function. The estimated particle size of all ferrite compositions derived from TEM observations was roughly twice the average crystallite size derived from X-ray diffraction measurements, as can be seen in the figure. The average particle size of the same ferrite compositions produced by the coprecipitation method was nearly three-times larger than the average crystallite size, suggesting a similar trend [24]. This variation was attributed to the fact that X-ray diffraction measures the average crystallite size, whereas TEM measures the average particle size, which can be composed of multiple crystallites. As a result, the particle size must always be larger than the crystallite size.

### 3.3. FT-IR Analysis

**Figure 5:**
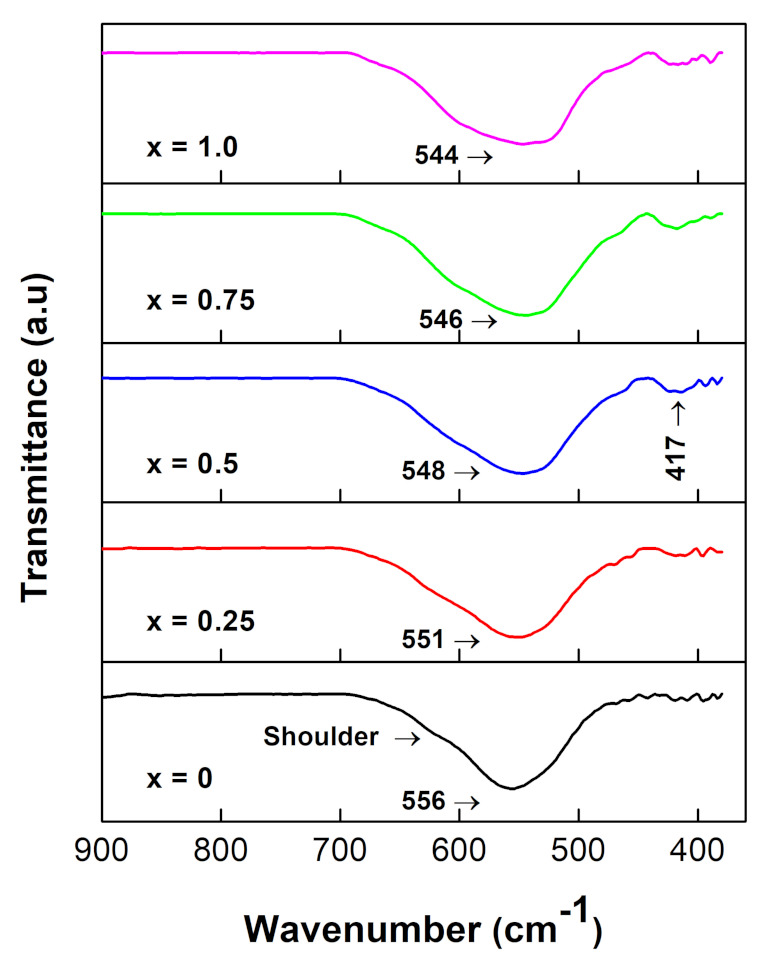
FT-IR spectra of the ${\mathrm{Ni}}_{x}{\mathrm{Co}}_{1-x}{\mathrm{Fe}}_{2}O_{4}$ spinel ferrites with 0≤x≤1.

The obtained FT-IR spectra for all ${\mathrm{Ni}}_{x}{\mathrm{Co}}_{1-x}{\mathrm{Fe}}_{2}O_{4}$ ferrites are shown in Figure 5. “For spinel ferrites, four modes of vibration are expected to occur in the $\nu_{1}\;\left(650-550\;{\mathrm{cm}}^{-1}\right)$, $\nu_{2}\;\left(525-390\;{\mathrm{cm}}^{-1}\right)$, $\nu_{3}\;\left(380-335\;{\mathrm{cm}}^{-1}\right)$ and $\nu_{4}\;\left(300-200\;{\mathrm{cm}}^{-1}\right)$ regions” [5]. Two prominent metal–oxygen bands may be recognized in the figure at frequencies of 556 ${\mathrm{cm}}^{-1}$ and 417 ${\mathrm{cm}}^{-1}$. The higher frequency band was created by the stretching vibrations of the metal–oxygen bond in the tetrahedral sites. The metal–oxygen vibrations in the octahedral sites created the lower-frequency one. These two absorption bands confirmed that a single-phase spinel structure with two lattices centered at the tetrahedral and octahedral sites was formed [10]. The tetrahedral region in all ferrite samples had a shoulder peak at ∼600 ${\mathrm{cm}}^{-1}$, suggesting that the tetrahedral sites were most often occupied by more than one type of cation, as shown in the figure. As a result, the ferrite nanoparticles produced were very likely to have mixed spinel-type structure. As Ni content increased, the high-frequency peak shifted slightly to the low-frequency regions: 556 cm${}^{-1}$, 551 cm${}^{-1}$, 548 cm${}^{-1}$, 546 cm${}^{-1}$, 544 cm${}^{-1}$. The last two absorption bands ($\nu_{3}$ and $\nu_{4}$) were not visible because they were outside the wave number range of the FT-IR machine that was used to collect the FT-IR absorption spectra.

### 3.4. Magnetic Properties

#### 3.4.1. M vs. H Curves

**Figure 6:**
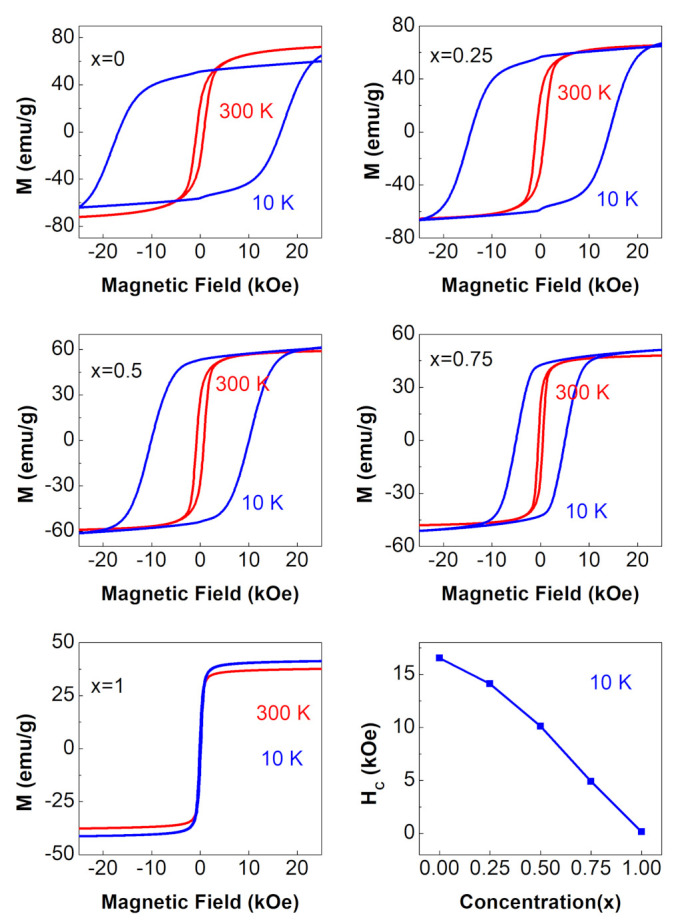
Magnetic hysteresis loops for the ${\mathrm{Ni}}_{x}{\mathrm{Co}}_{1-x}{\mathrm{Fe}}_{2}O_{4}$ ferrites with 0≤x≤1. The figure in the lower right corner shows the variation of $H_{c}$ (at 10 K) with Ni content.

The magnetic hysteresis loops of the ${\mathrm{Ni}}_{x}{\mathrm{Co}}_{1-x}{\mathrm{Fe}}_{2}O_{4}$ ferrites with 0≤x≤1 are shown in Figure 6. These loops were recorded in a magnetic field of up to 25 kOe at various temperatures. All ferrite samples were in a single ferrimagnetic phase between 300 K and 10 K, as shown in the figure. The RT coercivity of the ferrites was observed to decrease from ($H_{c}$∼770 Oe) for cobalt ferrite (x = 0) to ($H_{c}$∼42 Oe) for nickel ferrite (x = 1). This was expected because cobalt ferrite is a hard ferromagnetic material characterized by high coercivity, whereas nickel ferrite is a soft ferromagnetic material characterized by low coercivity. These measured values of RT coercivity of the end samples were consistent with the reported values of 890 Oe for cobalt ferrite and 50 Oe for nickel ferrite [25]. It is also clear from the figure that ferrimagnetism of ferrite nanoparticles with 0≤x≤0.75 was strongly temperature dependent. The $H_{c}$ of ${\mathrm{CoFe}}_{2}O_{4}$ (∼17 kOe) was substantially higher than that of bulk ${\mathrm{CoFe}}_{2}O_{4}$ (6.8 kOe) at 10 K [26], indicating that these ferrite nanoparticles were truly single-domain nanoparticles. The small value of $H_{c}$ of the ${\mathrm{NiFe}}_{2}O_{4}\;\left(x=1\right)$ ferrite and its very weak temperature dependence suggested that this sample was in a multi-domain regime. Furthermore, at 10 K, as seen in the lower right corner of Figure 6, the coercivity of all ferrite samples reduced with increasing Ni concentration, going from ∼17 kOe to ∼0.15 kOe as x increased from 0 to 1. As ions of the hard magnetic material (Co) were continuously replaced by ions of the soft magnetic material (Ni), such a reduction in coercivity was to be expected.

#### 3.4.2. LA Approach

When a large magnetic field of 25 kOe was used, Figure 6 clearly illustrates that the magnetization was not saturated. As a consequence, we determined the saturation magnetization ($M_{s}$) and the first anisotropy constant ($K_{1}$) using the law of approach to saturation (LA). “This approach is typically good for describing magnetization near saturation and is valid at fields much higher than the coercive field ($H\gg H_{c}$) [5]. The magnetization (M) near the saturation is determined by:

(2) $$M=M_{s}\;\left(1-\frac{b}{H^{2}}\right)+\kappa H$$

where $b=\frac{8}{105}\frac{K_{1}^{2}}{\mu_{0}^{2}M_{s}^{2}}$, $M_{s}$ is the saturation magnetization, H is the applied field, $\mu_{0}$ is free-space magnetic permeability, and $K_{1}$ is the first anisotropy constant (also known as the cubic anisotropy constant)” [5]. The κH on the right is frequently referred to as a paramagnetism-like term [27]. This term was found to be required in order to fit the obtained data.

**Figure 7:**
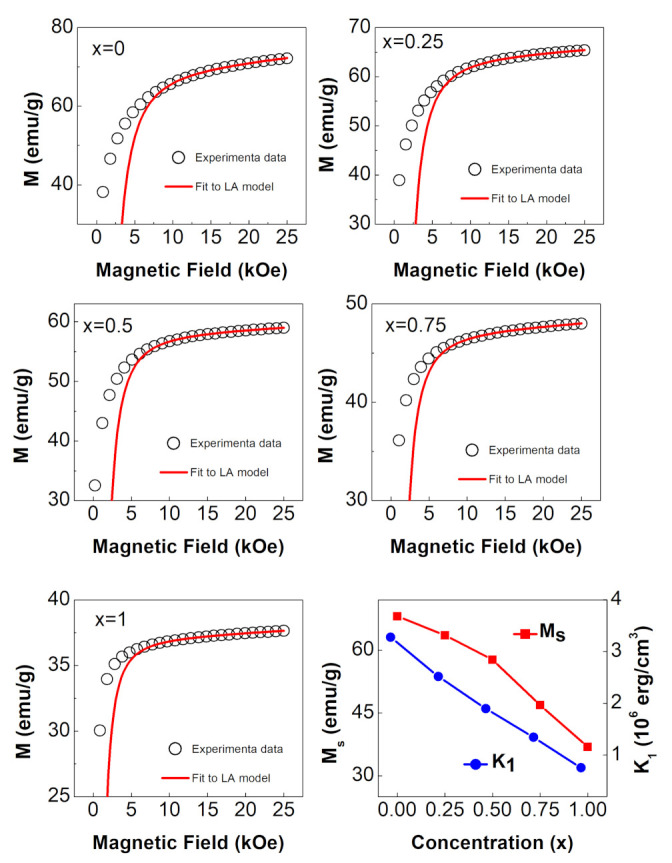
Room temperature magnetization curves of the ${\mathrm{Ni}}_{x}{\mathrm{Co}}_{1-x}{\mathrm{Fe}}_{2}O_{4}$ ferrites with 0≤x≤1, fit to LA model. The figure in the lower right corner shows the variation of $M_{s}$ and $K_{1}$ with Ni content.

The RT magnetic hysteresis loops of the ${\mathrm{Ni}}_{x}{\mathrm{Co}}_{1-x}{\mathrm{Fe}}_{2}O_{4}$ ferrites with 0≤x≤1 and the accompanying LA fits are shown in Figure 7. Since the LA model accurately describes magnetization at high fields, the fit was limited to magnetization data spanning 10 kOe to 25 kOe, as shown in the figure. The $M_{s}$ and $K_{1}$ values derived from the fit, as well as the $H_{c}$ and $M_{r}$ values are shown in Table 3. The variation of $M_{s}$ and $K_{1}$ with Ni content is depicted in the lower right corner of Figure 7. Both quantities were observed to decrease as Ni content increased. The decrease in $M_{s}$ was due to the replacement of higher-magnetic-moment ($3\mu_{B}$) Co^2+^ ions with lower-magnetic-moment ($2\mu_{B}$) Ni^2+^ at the octahedral sites. The obtained $M_{s}$ values for ${\mathrm{CoFe}}_{2}O_{4}$ ferrite (68.1 emu/g) and ${\mathrm{NiFe}}_{2}O_{4}$ ferrite (36.9 emu/g) were found to be lower than the reported values of 93 emu/g and 55 emu/g for their bulk counterparts [23]. The presence of a magnetically dead layer on the surface of the nanoparticles, the presence of canted spins, or the presence of a spin-glass-like behavior of surface spins can all be attributed to the decrease in magnetization of these nanoparticles [28]. The decrease in $K_{1}$ was attributed to the replacement of higher-anisotropy Co^2+^ ions having three unpaired electrons ($\left[\mathrm{Ar}\right]3d^{7}4s^{2}$) with lower-anisotropy Ni^2+^ ions having two unpaired electrons ($\left[\mathrm{Ar}\right]3d^{8}4s^{2}$). The measured values of $K_{1}$ for ${\mathrm{CoFe}}_{2}O_{4}$ ferrite ($3.28\times 10^{6}$$\mathrm{erg}/{\mathrm{cm}}^{3}$) and ${\mathrm{NiFe}}_{2}O_{4}$ ferrite ($0.75\times 10^{6}$$\mathrm{erg}/{\mathrm{cm}}^{3}$) were consistent with the reported values of ($3.8\times 10^{6}$$\mathrm{erg}/{\mathrm{cm}}^{3}$) [29] and ($0.9\times 10^{6}$$\mathrm{erg}/{\mathrm{cm}}^{3}$) [12] for ${\mathrm{CoFe}}_{2}O_{4}$ and ${\mathrm{NiFe}}_{2}O_{4}$, respectively. The squareness ratio, $M_{r}/M_{s}$, values are displayed in Table 3. These values were consistent with the reported values of 0.36, 0.48, 0.47, 0.61, and 0.18 for corresponding concentrations of x = 0, 0.25, 0.5, 0.75, and 1 for single-domain ${\mathrm{Ni}}_{x}{\mathrm{Co}}_{1-x}{\mathrm{Fe}}_{2}O_{4}$ ferrite nanoparticles prepared by the hydrothermal method [25]. When the squareness ratio fell below 0.6, the prepared nanoparticles were said to be in the single-domain regime [30]. The LA model was only implemented for magnetization data at 300 K. When applied to low temperature data, particularly at 10 K, this model did not produce good results because the coercive field had a higher value than at 300 K, resulting in the validity condition ($H\gg H_{c}$) not being met [5].

The distribution of cations between the A and B sites had a substantial impact on the magnetic properties of spinel ferrites. For inverse spinel ferrites, the distribution took the form ${\left({\mathrm{Fe}}^{3+}\right)}_{A}{\left[{\mathrm{Ni}}_{x}^{2+}{\mathrm{Co}}_{1-x}^{2+}\;{\mathrm{Fe}}^{3+}\right]}_{B}\;O_{4}^{2-}$. The magnetic moments of ions occupying the A and B sites were opposite each other, according to Neel’s two sublattice model of ferrimagnetism [24]. Therefore, the net magnetization was $n_{B}\left(x\right)=M_{B}\left(x\right)-M_{A}\left(x\right)$, where $M_{B}\left(x\right)$ and $M_{A}\left(x\right)$ are the B and A sublattice magnetic moments in $\mu_{B}$[5]. The magnetic moments of ${\mathrm{Fe}}^{3+}$, ${\mathrm{Co}}^{2+}$, and ${\mathrm{Ni}}^{2+}$ had ideal values of $5\mu_{B}$, $3\mu_{B}$, and $\;2\mu_{B}$, respectively. Based on these values, the net magnetic moments were calculated and shown in Table 4. On the other hand, the measured values of the magnetic moments can be found using “$n_{B}=\frac{A\times M_{S}}{5585}$, where A is molecular weight in g/mol, $M_{s}$ is saturation magnetization in emu/g, and 5585 is the magnetic factor” [23]. Using the last equation, the measured net magnetic moments are shown in Table 4 as well. It is obvious that the measured magnetic moments were somehow consistent with the calculated values from Neel’s two-sublattice model, suggesting that Co^2+^ ions on the octahedral sites were successfully replaced by Ni^2+^ ions, and hence, the overall magnetization was reduced. The small variations between the calculated and measured values of magnetic moments can be related to the existence of tiny amounts of Ni^2+^ or Co^2+^ ions in the tetrahedral sites and/or to the possibility of a tiny amount of Fe^3+^ ions migrating from the octahedral sites to the tetrahedral sites, suggesting that these nanoparticles adopted a mixed-spinel-type structure rather than an ideal-inverse-spinel-type structure. This is consistent with the appearance of the shoulder peak overlapping with the main tetrahedral peak in the FT-IR spectra (see Figure 5).

#### 3.4.3. M vs. T Curves

**Figure 8:**
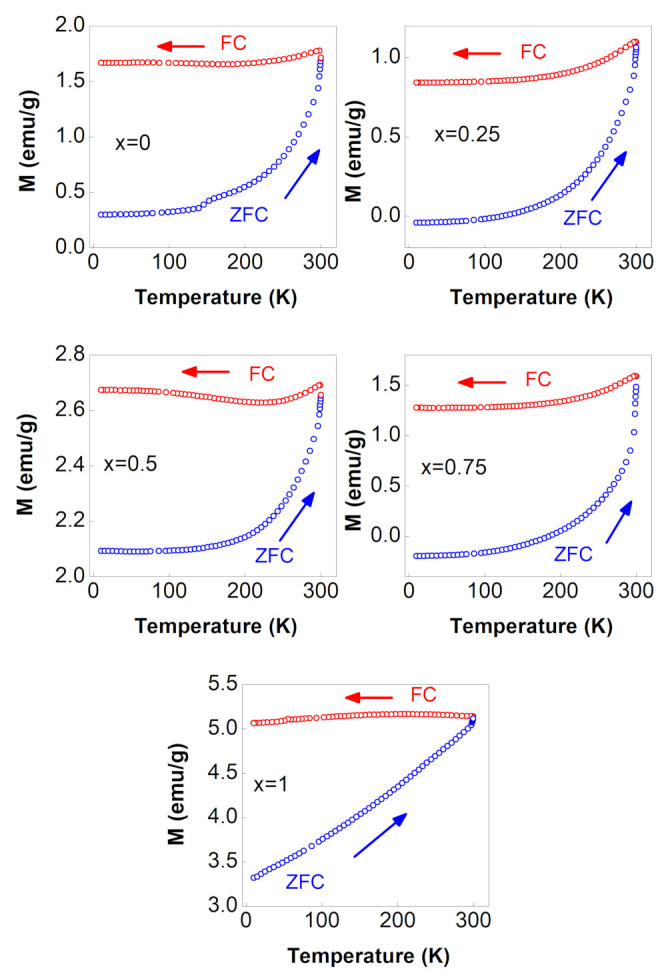
ZFC–FC magnetization curves of the ${\mathrm{Ni}}_{x}{\mathrm{Co}}_{1-x}{\mathrm{Fe}}_{2}O_{4}$ ferrites with 0≤x≤1, recorded with a magnetic field of 100 Oe.

The ZFC–FC magnetization curves were obtained using a standard method explained in our recent paper [12]. The ZFC–FC magnetization curves of ${\mathrm{Ni}}_{x}{\mathrm{Co}}_{1-x}{\mathrm{Fe}}_{2}O_{4}$ ferrites with 0≤x≤1 recorded in a tiny magnetic field of 100 Oe are shown in Figure 8. The figure shows that all ferrite samples were in a single ferrimagnetic phase across the entire temperature range. The corresponding M–H curves measured at 300 K and 10 K support this (see Figure 6). The lack of a peak in all ZFC curves indicated that the blocking temperature ($T_{B}$) was considerably higher than room temperature. The FC curves were nearly flat, indicating spin-glass behavior caused by interactions between ferrimagnetic nanoparticles and/or random freezing of surface spins [5,12]. “A spin glass is a collection of interacting magnetic moments in which the sign and magnitude of these interactions are randomly distributed” [31]. The unsystematic behavior of the magnetization values of all FC curves at a zero temperature limit demonstrated this randomness. As shown in the figure, extrapolating the FC magnetization curves to the 0 K limit demonstrated that there was no systematic behavior of the FC magnetization values with Ni content.

## 4. Conclusions

The hydrothermal method was employed successfully to prepare ${\mathrm{Ni}}_{x}{\mathrm{Co}}_{1-x}{\mathrm{Fe}}_{2}O_{4}$ ferrite nanoparticles with 0≤x≤1 and sizes less than 40 nm. The results of Rietveld refinement indicated that all prepared ferrites were in a single phase with a spinel-type structure and that increasing the Ni content reduced the lattice parameter from 8.3706 Å for ${\mathrm{CoFe}}_{2}O_{4}$ (x=0) to 8.3303 Å for ${\mathrm{NiFe}}_{2}O_{4}$ (x=1). The average crystallite size and X-ray density were found to increase with increasing Ni content from 17.4 nm and 5.31 $g/{\mathrm{cm}}^{3}$ to 41.4 nm and 5.39 $g/{\mathrm{cm}}^{3}$ as x increased from 0 to 1. According to TEM examinations, the nanoparticles were found to be spherical in shape, indicating high crystallinity caused by thermal annealing. The presence of a single-phase spinel-type structure with two sublattices was confirmed by the FT-IR analysis. The magnetization isotherms showed that over a wide range of temperature, all nanoparticles were found in a single ferrimagnetic phase and that Ni substitution decreased the ferrimagnetism. $M_{s}$ and $K_{1}$ were observed to decrease with increasing Ni content from 68.1 emu/g and $3.28\times 10^{6}$$\mathrm{erg}/{\mathrm{cm}}^{3}$ to 36.9 emu/g and $0.75\times 10^{6}$$\mathrm{erg}/{\mathrm{cm}}^{3}$ as x increased from 0 to 1. The big difference in $H_{c}$ of the x = 0, 0.25, 0.5, 0.75 ferrites between 300 K and 10 K indicated that these ferrite nanoparticles were truly single-domain nanoparticles. The ZFC–FC curves revealed the existence of spin-glass-like behavior in these ferrite nanoparticles over the entire temperature range.

## Figures and Tables

**Table 1:** Rietveld refinement input parameters for ${\mathrm{Ni}}_{x}{\mathrm{Co}}_{1-x}{\mathrm{Fe}}_{2}O_{4}$ ferrite nanoparticles with 0≤x≤1. The description of the parameters is given in the text.

x	Atom	(8a)	(16d)	(32e)	sof
		xyz	xyz	xyz	
**0**	**Co**		0.50.50.5		0.5
	**Fe**		0.50.50.5		0.5
	**Fe**	0.1250.1250.125			1
	**O**			0.250.250.25	1
**0.25**	**Ni**		0.50.50.5		0.125
	**Co**		0.50.50.5		0.375
	**Fe**		0.50.50.5		0.5
	**Fe**	0.1250.1250.125			1
	**O**			0.250.250.25	1
**0.5**	**Ni**		0.50.50.5		0.25
	**Co**		0.50.50.5		0.25
	**Fe**		0.50.50.5		0.5
	**Fe**	0.1250.1250.125			1
	**O**			0.250.250.25	1
**0.75**	**Ni**		0.50.50.5		0.375
	**Co**		0.50.50.5		0.125
	**Fe**		0.50.50.5		0.5
	**Fe**	0.1250.1250.125			1
	**O**			0.250.250.25	1
**1**	**Ni**		0.50.50.5		0.5
	**Fe**		0.50.50.5		0.5
	**Fe**	0.1250.1250.125			1
	**O**			0.250.250.25	1

**Table 2:** Structural parameters and Rietveld agreement R-factors for the ${\mathrm{Ni}}_{x}{\mathrm{Co}}_{1-x}{\mathrm{Fe}}_{2}O_{4}$ ferrites with 0≤x≤1. The description of the parameters is given in the text.

x	0	0.25	0.5	0.75	1
**D (nm)**	17.4	21.1	26.1	31.7	41.7
$d_{x}\;\left(g/{\mathrm{cm}}^{3}\right)$	5.31	5.34	5.36	5.38	5.39
*R-factors* (%)					
$R_{\exp}$	1.261	1.378	1.402	1.454	1.414
$R_{\mathrm{wp}}$	1.303	1.444	1.439	1.528	1.788
GoF ($\chi^{2}$)	1.07	1.1	1.05	1.1	1.6
*Cell parameters* (Å)					
** $a_{\exp}$ **	8.3706	8.3539	8.3458	8.3345	8.3303
**u**	0.2564	0.2667	0.2691	0.2698	0.2565
$R_{A}$	1.9051	2.0807	2.0266	1.9214	1.8986
$R_{B}$	2.0405	1.9441	1.9678	2.0184	2.0298

**Table 3:** $H_{c}$, $M_{r}$, $M_{s}$, $M_{r}/M_{s}$, and $K_{1}$ of the ${\mathrm{Ni}}_{x}{\mathrm{Co}}_{1-x}{\mathrm{Fe}}_{2}O_{4}$ ferrites with 0≤x≤1 measured at 300 K. The description of the parameters is given in the text.

*x*	$\;\;\;\;\;\;\;\;\;\;H_{c}$	$\;\;\;\;\;\;\;\;\;\;M_{r}$	$\;\;\;\;\;\;\;\;\;\;M_{s}$	$M_{r}/M_{s}$	$K_{1}$
	(Oe)	(emu/g)	(emu/g)		$\;\;\;\;(\mathrm{erg}/{\mathrm{cm}}^{3})\;\;\;\;\;\;$
**0**	770	24.1	68.1	0.35	$3.28\times 10^{6}$
**0.25**	802	27.7	63.6	0.44	$2.52\times 10^{6}$
**0.5**	649	27.4	57.7	0.47	$1.89\times 10^{6}$
**0.75**	390	21.7	46.8	0.46	$1.34\times 10^{6}$
**1**	42.1	4.6	36.9	0.12	$0.75\times 10^{6}$

**Table 4:** Molecular weight (A), RT saturation magnetization ($M_{s}$), calculated net magnetic moment $n_{B}\left(\mathrm{calculated}\right)$, and measured net magnetic moment $n_{B}\left(\mathrm{measured}\right)$ of the ${\mathrm{Ni}}_{x}{\mathrm{Co}}_{1-x}{\mathrm{Fe}}_{2}O_{4}$ ferrites with 0≤x≤1.

*x*	A	$\;\;\;\;\;\;\;\;\;\;M_{s}$	$\;\;\;\;\;\;\;\;\;\;n_{B}\left(\mathrm{calculated}\right)$	$n_{B}\left(\mathrm{measured}\right)$
	(g/mol)	(emu/g)	$\;\;\;\;\;\;\;\;\;\;\;\;\;\;\;\;\;\;(\mu_{B})\;\;\;\;\;\;$	$\;\;\;\;\;\;\;\;\;\;\;\;\;\;\;\;(\mu_{B})\;\;\;\;\;\;$
**0**	234.63	68.1	3	2.86
**0.25**	234.57	63.6	2.75	2.67
**0.5**	234.51	57.7	2.5	2.42
**0.75**	234.45	46.8	2.25	1.96
**1**	234.39	36.9	2	1.55

## Data Availability

The data used to support the findings of this study are included within the article.
